# Cohort study on educational well-being of children of Chinese origin adopted into transracial and international families in the Spanish education system

**DOI:** 10.12688/f1000research.52409.2

**Published:** 2022-02-17

**Authors:** David Doncel-Abad, Pablo Cabrera-Álvarez

**Affiliations:** 1 Sociology and Communication, University Salamanca, Salamanca, 37007, Spain; 2 Sociology and Communication, University of Salamanca, Salamanca, 37007, Spain

**Keywords:** Well-being, transnational adoption, race and ethnicity

## Abstract

The following dataset focuses on the educational well-being of adopted girls of Chinese origin in Spanish schools. Due to its characteristics, the presence of this group may generate complex interaction dynamics in school; particularly regarding bullying in school linked to factors such as the acceptance of others. These are dynamics, which may indeed condition the educational experience of this social group. Therefore, the aim of creating this dataset is to measure the educational well-being of children of Chinese origin adopted into transracial families in Spain. Although this research was justified by the lack of studies on this social group, we aimed to go one-step further, we also studied the correlation between this social group’s educational experience, and to what extent they show an interest in Chinese culture. As we have written before, we incorporated the concept of Well-Being and we worked with the following indicators: Satisfaction with Life, Social Life, and Bullying in School, Racial Bullying, Personal Identity and Interest in Chinese Culture. To achieve the objective set forth, we conducted a questionnaire. The final sample consisted of 268 individuals. The creation of this dataset provided us with information that can shed light on the relationship between adoption, race/ethnicity and the educational experience of adopted children of Chinese origin.

## Introduction

The following study focuses on the social well-being or educational experience of adopted girls of Chinese origin in Spanish schools. At the end of the 1990s, Spain became the world’s largest adoption receiving country in the world. Geographically, Asia, and specifically China, was the prefered place among adoptive parents. According to data from the National Statistics Institute of Spain, the first girls adopted from China began to arrive in 1995. From that moment on, Chinese children soon became the most adopted of all: in 2005, out of 2,854 adopted children of Asian origin, 2,753 came from China. A decade later, it is estimated that there are approximately 18,000 adopted children of Chinese origin in Spain, which makes this the largest group when it comes to international adoptions.

This social group presents a distinctive characteristic, which is typical of adoptive families who are of dual nationality and ethnicity: on the one hand, they have Asian phenotypic traits from birth; yet on the other, they are part of the dominant culture due to adoption ( Grotevant & VonKorff, 2011). This characteristic, named “the transracial paradox” by Lee (2003), has contributed to the enrichment of social heterogeneity in the classrooms but has also increased the complexity of the relationships formed in the schools ( Kim & Lee, 2020). We must not forget that a key element to achieving a necessary and proper coexistence lies in the development of the relationships between the different members of the educational community.

Scientific literature on this matter has indicated that one factor linked to bullying in school is racial/ethnic prejudice ( Verkuyten & Thijs 2001, 2002, 2006). In particular, a correlation between Asian race or ethnicity and bullying in school has been identified by Juvonen, Grahan and Schsuter (2003) and Mouttapa, Valente, Gallaher, Rohrbach and Unger (2004). Admittedly, it is also indicated that racial/ethnic factor alone does not increase the likelihood of being victimized ( Díaz Aguado *et al.*, 2013), yet it is an influencing factor when linked to an array of material and/or social circumstances.

Few studies have been carried out on the analysis of racial/ethnic bullying in school together with the adoption factor in the case of transracial adoptive families. However, there have been significant contributions. Raaska *et al.* (2012) found that being adopted increases the chances of being bullied in school, but only when combined with other individual variables. Moran *et al.* (1993) proved that the “being Asian” factor has an impact when it comes to friendship or companionship compared to other social groups. As for the studies carried out by Adams, Tessler and Gamache (2005) and Tang and Arthur (2012), it was proven that if an educational environment offers a positive experience to adopted individuals belonging to transracial families, this contributes to the child’s positive acceptance of the uniqueness of their ethnic or racial origin, fosters their self-esteem and, consequently, their educational well-being. In Spain, Fernández (2016) pointed out that “being Chinese and adopted becomes ‘something bad’ and leads to insults or even bullying” (Fernández-Cáceres, 2016, p. 409). However, Gil, Doncel, Morales and Lambea (2020) revealed that these children may be mocked due to their Asian phenotypic traits, yet not for being adopted.

In summary, the group of children of Chinese origin adopted in Spain can be considered a group whose presence in schools may generate complex interactional dynamics. Particularly regarding bullying in school linked to factors such as the acceptance of the “other”. These are dynamics, which may indeed condition the educational well-being of this social group.

Consequently, this study aimed to create a dataset that presented the educational well-being of adopted children of Chinese origin in transracial families in Spain. This research was justified by the lack of studies on this social group, that are relevant to understand the conditions of this minority in Spanish schools.

## Methods

### Framework

The target population of this study was formed of teenagers adopted from the People’s Republic of China who were enrolled in Spanish schools. The initial age range was between 14 and 16 years (both ages included). All these teenagers must have arrived in Spain around 2003-2005, as the data collection started on the 2019-2020 school year.

According to the data offered by the Directorate-General for Social Policy, Families and Childhood, under the Ministry of Health, Social Policy and Equality (2010, p. 108), there were a total of 7,612 adoptions from China between 2003 and 2005, which makes this country the most notable when it comes to adoption numbers. Also, taking into account the fact that most adopted children are girls (around 97% according to the data from Castilla y León – a Spanish region; Fernández Cáceres, 2016; SSCYL, 2018), we could estimate that the number of girls of Chinese origin was around 7,380. The strict birth control measures and the one-child policy implemented in the People’s Republic of China, together with the patriarchal tradition of preferring sons to daughters, explains why girls were abandoned more than boys.

### Sampling procedure

As we were lacking a sampling framework, we decided to ask for cooperation from adoptive parents’ associations to be able to access the target population. We collected data through a census of parents who had joined adoption associations and who were able to complete an adoption process in China between 2003 and 2006. This approach is justified by the impracticability of screening the general population in order to locate homes with adopted children. Furthermore, this strategy is based on the evidence that a large number of parents who adopt children from China tend to join an association in order to go through this process with greater guarantees.

This strategy allowed us to access a large group of adopted children through their parents. In order to do so, we created an exhaustive list of adoptive parents’ associations registered in Spain: ADOPCHINA, AFADA, AFADENA, AFAAR, AFAHU, AFAIC, AFAMUNDI, AIBA, AMADA, ANICHI, ARFACYL, ASTURADOP, ATLAS, AKUNA INTERNACIONAL, ACI, Hijos que esperan, MANANIA, MUDAN, UME ALAIA, TRANSMES, Fundación CORA and AFAC. The research team requested these associations to forward our study to their members who met the requirements to be a part of the target population for this research.

The online questionnaire addressed to adopted children was sent by email to the contacts provided. We first sent the questionnaire on September 12, 2019 at 12:00 AM. The responses to the questionnaire were gathered over three months (from October 2 to December 17). While compiling the responses, we exhaustively monitored the sample and the established exclusion criteria.

The incidents we encountered while gathering the responses to the questionnaire were as follows:

• A questionnaire was deleted because it was duplicated.
• Two questionnaires were deleted because they were incomplete.

On December 17, we obtained the final sample, which consisted of *n* = 268 individuals between 9 and 19 years, both ages included. Of them, 98.1% were girls and 1.9% boys.

It is worth noting that there could have been some measurement bias due to the children filling in the questionnaire under their parents’ supervision. It is possible that the children felt inhibited or that their responses were not as precise because of their parents being present.

### Variables

In this study, we adopted the concept of Well-Being used by the Organization for Economic Cooperation and Development (OECD) in the PISA 2017 report in order to measure the educational well-being of this social group in schools. The OECD related the concept of students’ social well-being to a variety of factors: satisfaction with life, schoolwork-related anxiety (homework and tests), motivation to achieve, sense of belonging at school, bullying, and social status and inequalities. Taking into account these elements influencing the concept of Educational Well-being, and in accordance with the purpose of this study, we have looked at the following factors: Satisfaction with Life, Social Life *,* Bullying in School, Racial/Ethnic Bullying, Personal Identity and Interest in Chinese Culture.

• *Satisfaction with Life* measures the degree of subjective well-being of students in relation to their own lives. In the questionnaire used in this study, the interviewees’ satisfaction was rated on a scale of 0 to 10, 0 being the lowest satisfaction and 10 the highest.
• *Social Life at School* (α = .81) has been defined as social integration or social relationships that the individual has established with his or her classmates ( OECD, 2017). The items used to measure the degree of social integration were: “We get along well in the classroom,” “I express and defend my opinions without hurting others,” “I feel that I have friends,” “My classmates care about me when I need them,” “I like to collaborate with my friends,” “My classmates like me,” “I’d rather work in teams than alone,” “I felt different from the rest,” “It is hard for me to express my feelings.” Each indicator was rated on a Likert scale, ranging from 1 (Strongly Disagree) to 5 (Strongly Agree). Based on these indicators, we created the Social Life Scale, the scale being from 1 (low degree of social integration at school) to 5 (high degree of social integration).
• *Bullying* (α = .72) has been defined as “an intentional, repeated, negative (unpleasant or hurtful) behavior by one or more persons directed against a person who has difficulty defending himself or herself” (Olweus, 2006, p. 81). In this research, this scope was studied by asking the typical questions to measure bullying in PISA ( OECD, 2017): “I have been physically hurt by other students,” “Other students took my things from me,” “Other students insulted me/called me names,” “Other students lied about me, talked behind my back or spread rumors about me,” “I was threatened by other students,” “Other students left me out of things/isolated me on purpose,” “Other students destroyed my things” and “Other students made fun of me.” Each indicator was measured on a Likert scale (1-Never, 2-Rarely, 3-Sometimes, 4-Frequently and 5-Always). From these indicators, we created the Bullying Scale, ranging from 1 (low perception of having experienced bullying) to 5 (high perception of having experienced bullying).
• *Racial/Ethnic Bullying* was studied by adding a racial element to the typical questions to measure bullying: “I felt intimidated, rejected or bullied because of my facial features.” This indicator was measured on a Likert scale (1-Never, 2-Rarely, 3-Sometimes, 4-Frequently and 5-Always).
• *Personal Identity*: In order to measure children’s perception of identity, we used one of the Social Life indicators: “I felt different from others.” This item was measured on a Likert scale, 1 being Strongly Disagree (greater integration) and 5 being Strongly Agree (little integration).
• *Interest in Chinese Culture* (α = .88), measured by the following indicators: “News about China interests me,” “I’d like to learn Chinese,” “I’d like my parents to go to Chinese restaurants more often,” “I’d like to attend Chinese celebrations,” “I’d like to know how non-adopted Chinese children live in my town,” “I’d like my parents to take me to China” and “I’d like my school to organize activities so I can learn about Chinese culture.” Each item was measured on a Likert scale, 1 being Strongly Disagree and 5 Strongly Agree. Based on these indicators, we created the Interest in Chinese Culture Scale, the scale ranging from 1 (little interest in learning about Chinese culture) to 5 (strong interest).

We added a set of sociodemographic variables to the analysis, these being:

• ISCED: Scale to measure the parents’ highest completed level of education, 1 being completed secondary education or lower and 5 being university education (master’s degree or doctorate).
• Age: recoded in four intervals: 9-13; 14-15; 16-17 and 18-19 years old.
• Age of arrival in Spain, measured in months.
• Type of school: Público (Public School), Concertado (Charter School), Privado (Private School)
• Sex.

## Composition of the final dataset

The final sample ( *n* = 268) consisted of a larger number of participants than expected from the initial sample design. Also, a broader part of the Spanish territory was represented. However, the sample criteria established for Castilla y León, which was the region we were interested in, were not met. We obtained data from adopted children of Chinese origin from the following Autonomous Communities (see Table 1):

**Table 1.** Distribution of the sample in autonomous communities in Spain.

	Cases	%
Andalucía	30	11.2
Aragón	13	4.9
Canarias	2	.7
Cantabria	7	2.6
Castilla La-Mancha	19	7.1
Castilla y León	72	26.9
Cataluña	12	4.5
Comunidad de Madrid	48	17.9
Comunidad Foral de Navarra	3	1.1
Comunidad Valenciana	9	3.4
Extremadura	17	6.3
Galicia	15	5.6
Islas Baleares	2	.7
La Rioja	7	2.6
País Vasco	5	1.9
Principado de Asturias	7	2.6
Total	268	100.0

In the following table ( Table 2), we can observe the profile of the final sample by sociodemographic variables:

**Table 2.** Sample profile by sociodemographic variables.

		Cases	%
**Sex**	Male	5	1.9
	Female	263	98.1
**Age of arrival in Spain**	Less than 1 year old	72	26.9
	From 1 to 2 years old	135	50.4
	2 years old or more	61	22.8
**Current education level**	Elementary Education	4	1.5
	Middle School	123	46.1
	High School	103	38.6
	Vocational Education	20	7.5
	University	17	6.4
**Type of school**	Public School	188	70.1
	Charter School	62	23.1
	Private School	18	6.7
**Parents’ highest completed level of education**	Middle School or lower	11	4.2
	Vocational Ed.: medium-level	15	5.7
	High School	26	9.8
	Vocational Ed.: higher-level	14	5.3
	University degree	168	63.6
	Postgraduate	30	11.4

## Ethical approval

Since the sample would be partially made up of minors, we requested the approval of the Bioethics Committee from the University of Salamanca. After checking that an ethical collection of minors data was guaranteed, the Bioethics Committee approved this project (ID: 390) on September 30, 2019.

## Consent

To carry out this project, before collecting the questionnaires, we obtained the informed consent form prior to the participants or their parents, in cases of minors, taking part in the research.

## Data availability

The data collected for this study cannot be made available to the public in an open repository since the parents, who consented before the children responded to the questionnaire, were informed that the data would be encoded, anonymized and stored in the Archives of the Faculty of Social Science of Salamanca—and that they would only be used for the purposes of this project. It was also agreed that, if any other researchers wished to access this database, they would have to contact the Faculty of Social Science of the University of Salamanca.

This project’s database is stored in the Archives of the Faculty of Social Science of the University of Salamanca, located in Campus Unamuno s/n, Salamanca 37006, Spain. This space can only be accessed by the members of the Deanery and the University Secretariat. If a third party wished to access this material, they would need to ask the Archives authorities for the corresponding authorization. Contact details: davidoncel@usal.es Telephone 923294500 Ext. 3113. All the information derived from this study will be used exclusively for the purposes indicated in the initial project.
