# Supplementary material for: Cohort study on educational well-being of children of Chinese origin adopted into transracial and international families in the Spanish education system
Source: F1000Res. 2022 Feb 17;10:495. Originally published 2021 Jun 25. [Version 2] doi: 10.12688/f1000research.52409.2 (PMC8861466; doi:10.12688/f1000research.52409.2)
Supplement: Peer review history

# Peer review: Reviewer response for version 2

**DOI:** 10.5256/f1000research.121379.r124126

**Published:** 2022-02-21

**Authors:** José David Urchaga-Litago

**Affiliations:** 1 Faculty of Communication, Pontifical University of Salamanca, Salamanca, Spain; 2 Faculty of Psychology, International University of La Rioja, Logroño, Spain

Recommendations have been included.

Are sufficient details of methods and materials provided to allow replication by others?

Yes

Is the rationale for creating the dataset(s) clearly described?

Yes

Are the datasets clearly presented in a useable and accessible format?

Partly

Are the protocols appropriate and is the work technically sound?

Yes

Reviewer Expertise:

Methodology and Statistics in Social and Human Sciences

I confirm that I have read this submission and believe that I have an appropriate level of expertise to confirm that it is of an acceptable scientific standard.

# Peer review: Reviewer response for version 1

**DOI:** 10.5256/f1000research.55683.r100286

**Published:** 2022-02-11

**Authors:** Kwok Ng

**Affiliations:** 1 Physical Activity for Health, Health Research Institute, Limerick, Ireland; 2 University of Eastern Finland, Kuopio, Finland

This is a nicely reported data set with rationale and background to the sample. Furthermore, given the limitations of the sampling frame, the authors have done a good job to locate such a diverse range of participants with similar characteristics of interest. 

 The data availability is of course a limitation, but the authors have given information for how to retrieve it, and although it has not been attempted, it is hoped that it would not impede on the progress of open science. 

 The variables are slightly limited, given the efforts taken to collect data from the sample, and a more holistic perspective of health may have been warranted. None the less, this is a starting point to understand the exposure to victimisation and self-perceptions around this matter from the perspective of Chinese adopted girls in Spain.

Are sufficient details of methods and materials provided to allow replication by others?

Yes

Is the rationale for creating the dataset(s) clearly described?

Yes

Are the datasets clearly presented in a useable and accessible format?

Partly

Are the protocols appropriate and is the work technically sound?

Yes

Reviewer Expertise:

public health and health promotion.

I confirm that I have read this submission and believe that I have an appropriate level of expertise to confirm that it is of an acceptable scientific standard.

# Peer review: Reviewer response for version 1

**DOI:** 10.5256/f1000research.55683.r100287

**Published:** 2021-11-29

**Authors:** José David Urchaga-Litago

**Affiliations:** 1 Faculty of Communication, Pontifical University of Salamanca, Salamanca, Spain; 2 Faculty of Psychology, International University of La Rioja, Logroño, Spain

- Abstract: Studies the incidence of bullying in school and satisfaction with life in a sample of children adopted from China.
- This study is very new in Spain. In addition, it is very important because adopted children of Chinese origin is the largest group.
- It is original to study “to what extent they show interest in Chinese culture”, but it is important to justify why that is, to briefly justify its theoretical importance. Neither is any result provided on this aspect. In this case, either present results or eliminate said objective (I recommend the latter).
- Well defined population and sample. Well measured variables. The authors are requested to reference the original studies of the measuring instruments. If they have been created "ad hoc" for this research, the authors must indicate it.
- Table 1: Eliminate the accumulated percentage.

**Important:** There is no Results section. Provide the study data about these conclusions:

1. The incidence of bullying in school is similar to the parameters observed in other reports (like PISA 2015) concerning the school population in Spain.
2. Moreover, this group stands out in particular due to its high level of satisfaction with life (in relation to expected results by age group compared to data from the PISA 2015 report for Spain) and its low perception of feeling different from others.

I do not know if the journal admits publishing studies without results, only presenting the conclusions. If so, I apologize for the last entry.

 I consider this to be a very important and original study. I think it should be indexed.

Are sufficient details of methods and materials provided to allow replication by others?

Yes

Is the rationale for creating the dataset(s) clearly described?

Yes

Are the datasets clearly presented in a useable and accessible format?

Partly

Are the protocols appropriate and is the work technically sound?

Yes

Reviewer Expertise:

Methodology and Statistics in Social and Human Sciences

I confirm that I have read this submission and believe that I have an appropriate level of expertise to confirm that it is of an acceptable scientific standard, however I have significant reservations, as outlined above.

# Response

**Published:** 2022-02-15

**Authors:** David Doncel-Abad

**Affiliations:** University of Salamanca, Spain

First, we want to thank the reviewer for his useful comments that have been fundamental to refining the final version of the paper. Moreover, we would like to add some clarifications to some of your questions and highlight the changes introduced in the new version of the paper.

 We adapted the statement about the research objectives, which are broader than assessing to what extent “the Chinese adopted children show interest in Chinese culture”. This secondary objective will be developed and assessed in other publications in the frame of the research project. We understand that this statement could be misleading, and we have removed it from the paper.

 We have included the references of the measures used in the study.

 Regarding the absence of a results section: This is a data note in which we describe a dataset and inform about the conditions under which other researchers can access it. The substantive results of this research are presented in other papers that are under review at the moment. However, we agree with the reviewer that the comments about the conclusions should not be part of a data note, and, therefore, we have removed them from the paper.

 We improved the paper with further clarifications in the methods section and style changes.
